# Dissemination and Effectiveness of the Peer Marketing and Messaging of a Web-Assisted Tobacco Intervention: Protocol for a Hybrid Effectiveness Trial

**DOI:** 10.2196/14814

**Published:** 2019-07-23

**Authors:** Jamie M Faro, Elizabeth A Orvek, Amanda C Blok, Catherine S Nagawa, Annalise J McDonald, Gregory Seward, Thomas K Houston, Ariana Kamberi, Jeroan J Allison, Sharina D Person, Bridget M Smith, Kathleen Brady, Tina Grosowsky, Lewis L Jacobsen, Jennifer Paine, James M Welch Jr, Rajani S Sadasivam

**Affiliations:** 1 Division of Health Informatics and Implementation Science Department of Population and Quantitative Health Sciences University of Massachusetts Medical School Worcester, MA United States; 2 Center for Healthcare Organization and Implementation Research Bedford Veterans Affairs Medical Center Bedford, MA United States; 3 Division of Biostatistics And Health Services Research Department of Population and Quantitative Health Sciences University of Massachusetts Medical School Worcester, MA United States; 4 Center of Innovation for Complex Chronic Healthcare Spinal Cord Injury Quality Enhancement Research Initiative Hines VA Medical Center Chicago, IL United States; 5 Department of Pediatrics Feinberg School of Medicine Northwestern University Evanston, IL United States; 6 S2S Patient Panel Worcester, MA United States; 7 Department of Psychiatry University of Massachusetts Medical School Worcester, MA United States

**Keywords:** smoking cessation, peer recruitment, digital Intervention, tailored, dissemination

## Abstract

**Background:**

Smoking continues to be the leading preventable cause of death. Digital Interventions for Smoking Cessation (DISCs) are health communication programs accessible via the internet and smartphones and allow for greater reach and effectiveness of tobacco cessation programs. DISCs have led to increased 6-month cessation rates while also reaching vulnerable populations. Despite this, the impact of DISCs has been limited and new ways to increase access and effectiveness are needed.

**Objective:**

We are conducting a hybrid effectiveness-dissemination study. We aim to evaluate the effectiveness of a machine learning–based approach (recommender system) for computer-tailored health communication (CTHC) over a standard CTHC system based on quit rates and risk reduction. In addition, this study will assess the dissemination of providing access to a peer recruitment toolset on recruitment rate and variability of the sample.

**Methods:**

The Smoker-to-Smoker (S2S) study is a 6-month hybrid effectiveness dissemination trial conducted nationally among English-speaking, current smokers aged ≥18 years. All eligible participants will register for the DISC (Decide2quit) and be randomized to the recommender system CTHC or the standard CTHC, followed by allocation to a peer recruitment toolset group or control group. Primary outcomes will be 7-day point prevalence and risk reduction at the 6-month follow-up. Secondary outcomes include recruitment rate, website engagement, and patient-reported outcomes collected via the 6-month follow-up questionnaire. All primary analyses will be conducted on an intent-to-treat basis.

**Results:**

The project is funded from 2017 to 2020 by the Patient Centered Outcomes Research Institute. Enrollment was completed in early 2019, and 6-month follow-ups will be completed by late 2019. Preliminary data analysis is currently underway.

**Conclusions:**

Conducting a hybrid study with both effectiveness and dissemination hypotheses raises some unique challenges in the study design and analysis. Our study addresses these challenges to test new innovations and increase the effectiveness and reach of DISCs.

**International Registered Report Identifier (IRRID):**

DERR1-10.2196/14814

## Introduction

Smoking continues to be a public health concern and is the leading cause of preventable death in the United States [1]. Annually, over 6 million deaths in the world are attributable to smoking, including 480,000 in the United States [1]. Although the overall rates of smoking have reduced, the rates among socioeconomically disadvantaged subgroups are considerably higher [2]. In particular, African American smokers suffer disproportionately due to smoking-related diseases including several cancers, cardiovascular disease, and cerebrovascular disease [2-4]. Although they smoke fewer cigarettes and start smoking at an older age, these smokers are more likely to die from smoking-related diseases than white smokers [1]. Identifying strategies to increase reach and effectiveness of tobacco cessation programs, especially among vulnerable populations, is an ongoing research challenge [5].

Digital Interventions for Smoking Cessation (DISCs) are health communication programs readily accessible via the internet and smartphones. DISCs can include a number of functions designed to support a smoker’s cessation attempt. Previous research has shown that DISCs can be effective. In our prior trial, our DISC—Decide2Quit [6]—achieved a cessation rate of 30% at 6 months, which is much higher than the 7% rate at which smokers quit without support [7]. DISCs have the potential to reach a large and diverse group of smokers. Access to DISCs has previously been limited for many smokers because of the disparities in internet access. However, the digital divide in internet access has decreased considerably with increased broadband availability and smartphone use [8,9]. Despite this increased access and potential for effectiveness, the impact of DISCs has been limited. New ways to increase the access and effectiveness of DISCs are needed.

In response to a Patient Centered Outcomes Research Institute (PCORI) call for communication and dissemination research, we proposed a dissemination study—Smoker-to-Smoker (S2S)—to test whether providing access to a peer recruitment toolset that facilitates recruitment of friends and family members to the intervention will increase recruitment rate and increase variability of the sample. Because of feedback from PCORI and peer review, we expanded our study to also test the effectiveness of a machine learning–based approach (recommender system) for computer-tailored health communication (CTHC). Thus, our project is a hybrid effectiveness dissemination trial including both effectiveness and dissemination hypotheses (Textbox 1). Conducting a hybrid study with both effectiveness and dissemination hypotheses raises some unique challenges in the design and analysis of our study. Our paper describes the intervention functions and the study protocol we developed to address previously mentioned challenges. We also describe the budget impact analysis we will use to assess the cost of implementing this intervention.

**Study hypotheses.**

Hypothesis 1: Dissemination

- H1A: Peer recruitment will recruit a greater proportion of African American smokers compared to standard online recruitment.
- H1B: Peer recruitment will reduce recruitment time (time to recruit each participant) compared to standard online recruitment.

Hypothesis 2: Repeated use of Decide2Quit functions

- H2A: Repeated use among those exposed to the fully enhanced group (access to peer recruitment toolset and recommender CTHC) will be greater than repeated use among those exposed to (1) peer recruitment toolset only, (2) recommender CTHC with no peer recruitment toolset, and (3) standard group (no peer recruitment toolset and standard CTHC).
- H2B: Repeated use among those exposed to the peer recruitment toolset will be greater than repeated use among those exposed to the standard group.
- H2C: Repeated use among those exposed to the recommender CTHC will be greater than repeated use among those exposed to the standard group.

Hypothesis 3: Effectiveness

- Quit rates and risk reduction among participants exposed to the recommender CTHC (A+B) will be greater than those among participants exposed to the standard group (C+D).

## Methods

### Study Overview

The goal of this study (ClinicalTrials.gov: NCT03224520) is to recruit 1200 smokers to test our effectiveness and dissemination hypotheses. To participate in the study, all smokers will register online for the Decide2Quit DISC. Randomization will occur after registration using a multilevel approach, as detailed below. We will follow smokers for 6 months from their registration date. Following a description of the intervention and comparison, our protocol is described in detail below. The protocol of our study was approved by the Institutional Review Board at the University of Massachusetts Medical School.

### The Smoker-to-Smoker Functions

#### The Computer-Tailored Health Communication System

CTHC is a frequently used tool in behavioral science and is focused on the selection of appropriate messages for an individual. CTHC increases personal relevance of health messaging by matching the messages to an individual’s or group’s characteristics [10]. CTHC can be effective in motivating behavior change [11-17]. Standard CTHC has traditionally been accomplished using rule-based approaches in which selected variables from patients’ baseline profile are matched to specific if-then tailoring rules to send tailored messages to specific subsets of patients [10,18]. As an alternative to rule-based approaches, companies such as Amazon use machine learning algorithms (ie, recommender systems) to tailor content. These recommender systems have several advantages over rule-based approaches, including the ability to continuously learn from user feedback (eg, liked product and products purchased) and enhance personal relevance. Textbox 2 provides an example of how a standard and recommender CTHC may differ in DISCs [18]. In our prior pilot randomized controlled trial (RCT) [19], we developed a recommender CTHC and compared this system with a standard CTHC system that showed effectiveness. The recommender system significantly outperformed the rule-based system on the number of days (out of 30) in which message relevance influenced smokers to quit. In the recommender system smokers, 74% strongly agreed or agreed that the messages influenced them to quit smoking, while this was only reported by 45% in the standard group (*P*<.01) [20]. Among those who completed follow-up, 36% (20/55) of the recommender system smokers and 32% (11/34) of the rule-based system smokers stopped smoking for one day or more (*P*=.70). Our goal in the S2S study is to rigorously test the recommender system against the standard CTHC for smoking cessation over a 6-month period. The primary difference between the recommender CTHC and standard CTHC will be the way in which messages are selected for the participant. Since our goal is to test the selection method, both systems will select from the same database of messages. We will first describe the motivational message database used in the study followed by the standard CTHC selection system and the recommender CTHC system.

**Computer health-tailored communication (CHTC). An example of a standard CHTC versus a recommender CHTC [ 17].**

John Smith, a 38-year-old smoker, has been smoking for 15 years. He has made multiple quit attempts in the past, but during each attempt, he gained between 10 and 20 pounds. Currently, fear of weight gain is a significant barrier to another quit attempt.

John is trying to quit again and registers on Decide2Quit. For 8 weeks, the system sends two tailored emails per week to John Smith to help him quit.

**Standard CTHC**

- In this approach, tailoring is based on information that John provides when he registers. For this example, we focus on one characteristic only: gender.
- Since women are typically more concerned about weight gain after quitting [21,22], experts have specified that half of the emails sent to women should contain information related to weight gain, but only one quarter of the emails sent to men should be focused on weight gain. 
- After registering on Decide2Quit, John receives the first email that targets weight gain support in the second week (third message) of the intervention. John likes the message and finds the tips it offers useful. He looks forward to receiving similar messages. However, the next five messages he receives focus on other topics. The next weight gain message arrives only on week 5.
- John does not think the system helped and fails in his attempt to quit.

**Recommender CTHC**

- In this approach, the selection of the message is based on the collective intelligence data, not on preset rules.  
- After registering on Decide2Quit, John visits the weight gain support page on the website (implicit data). The system uses these data and selects one of the messages targeting weight gain and sends it to John on week 2 (third message). John likes the messages and rates the message highly (explicit data). The system then notes both items of implicit and explicit feedback and regularly sends messages targeting weight gain to John. The system also repeats the message that John rates highly.
- Because the intervention targeted his needs more specifically, John finds these messages useful and succeeds in his attempt to quit.
- We have provided a simple example for ease of understanding. We have not included in this example how the group’s feedback can help John.

#### The Motivational Messaging Database

The messaging database includes 500 messages that were developed in our prior RCT, consisting of both expert-written messages and peer-written messages [23]. Expert-written messages were developed through an iterative expert group review process (behaviorists, physicians, and nurses). These messages were informed by current guidelines [24] and the Social Cognitive Theory [25], which incorporates vicarious learning, verbal persuasion, and expert messages that reflect the theoretical determinants of quitting such as positive outcome expectations and self–efficacy­-enhancing small goals [25]. Peer-written messages were written by current and former smokers responding to an online survey that presented four scenarios tailored by gender, age, and readiness-to-quit. These messages were then reviewed for use in our system. More details of our methodology to generate peer written messages have been previously published [23].

#### The Standard Computer-Tailored Health Communication System

Our comparison standard CTHC is a rule-based (if-then-else) system that tailors messages based on a smoker’s readiness to quit. For example, when a smoker logs on to Decide2Quit and indicates their readiness as “not ready to quit,” a message from those categorized for “not ready to quit” smokers will be picked at random and sent to the smoker. Similarly, if the smoker indicates their readiness as “set a quit date,” a message categorized for “set a quit date” smokers will be sent to the smoker. This system was tested in our prior study and demonstrated to be effective in increasing the 6-month smoking cessation by 9% (odds ratio [OR] 1.69, 95% CI 1.03-2.8) over a nonmessaging control [26]. Thus, our comparison will be a robust, active, and effective standard CTHC system.

#### Recommender Computer-Tailored Health Communication System

The details of our development and evaluation of the recommender system were previously published [20,26,27]. Briefly, we developed a hybrid recommender system that uses three input data sources to generate the recommendations, including metadata description of the messages, implicit feedback data, and explicit feedback data (smokers in the prior and current study). The recommender system consists of multiple components.

Our metadata includes a comprehensive coding of the messages. We developed these codes to facilitate further understanding of what did and did not work in these messages. These codes include constructs from multiple behavioral theories such as the Social Cognitive Theory, the Transtheoretical Model, and the Theory of Reasoned Action [28]. We also coded the messages for content that may be pertinent to a specific user, including health and lifestyle status, health issues, and treatment options. Overall, we developed 48 codes divided into 8 categories (General Treatments, Behavioral Treatments, Over the Counter and Prescription Treatments, Motivations, Health, Sociocultural Attributes, Author Attributes, and Author Interaction). Implicit feedback data are derived from user actions. As our implicit feedback data, we used the website return data of 900 smokers that participated in our prior RCT [19]. When an email was sent to these smokers, we tracked their website usage in the days following the email. Thus, we had data on the frequency at which each message promoted the use of Decide2Quit and the characteristics of the smokers that received these messages. Explicit feedback data consist of self-reported item ratings. We recruited 846 current or former smokers from online and local sources to rate the messages on the influence scale (see Data Collection and Outcomes). Each smoker was asked to rate 20 messages, resulting in 16,920 ratings. Several classic and state-of-the art collaborative filtering methods were evaluated for accurate prediction methods. The Bayesian Probabilistic Matrix Factorization (BPMF) was identified as the best single model in our evaluation and was used to develop the recommender CTHC. The BPMF model estimates a probability distribution over a joint embedding of users and items into complementary latent spaces. The rating a given user supplies for a given item is approximated by the expected value of the product of the latent user and item factor vectors representing the user-item pair, with the expectation taken over the uncertainty in embeddings [29]. In addition to explicit feedback ratings from smokers in prior studies, the recommender CTHC is programmed to use the explicit ratings of smokers receiving the messages (see Data Collection and Outcomes).

### Access to a Peer Recruitment Toolset

The primary element of the peer recruitment toolset is our Facebook website plugin [20,26,27]. The Facebook plugin will allow smokers to browse through their Facebook friends and recruit them by sending private recruitment messages. In our pilot study [20,26,27], providing smokers access to peer recruitment quadrupled our sample (190 smokers recruited 569 more smokers to the Decide2Quit DISC). Further, the smokers recruited by their peers were more likely to be African American as compared to those who were directly recruited from an online social network (23.8% vs 10.8%; *P*<.01 for all comparisons). Thus, in our dissemination hypothesis, we are testing if providing smokers access to peer recruitment specifically increases the proportion of African Americans in our sample.

**Figure 1:**
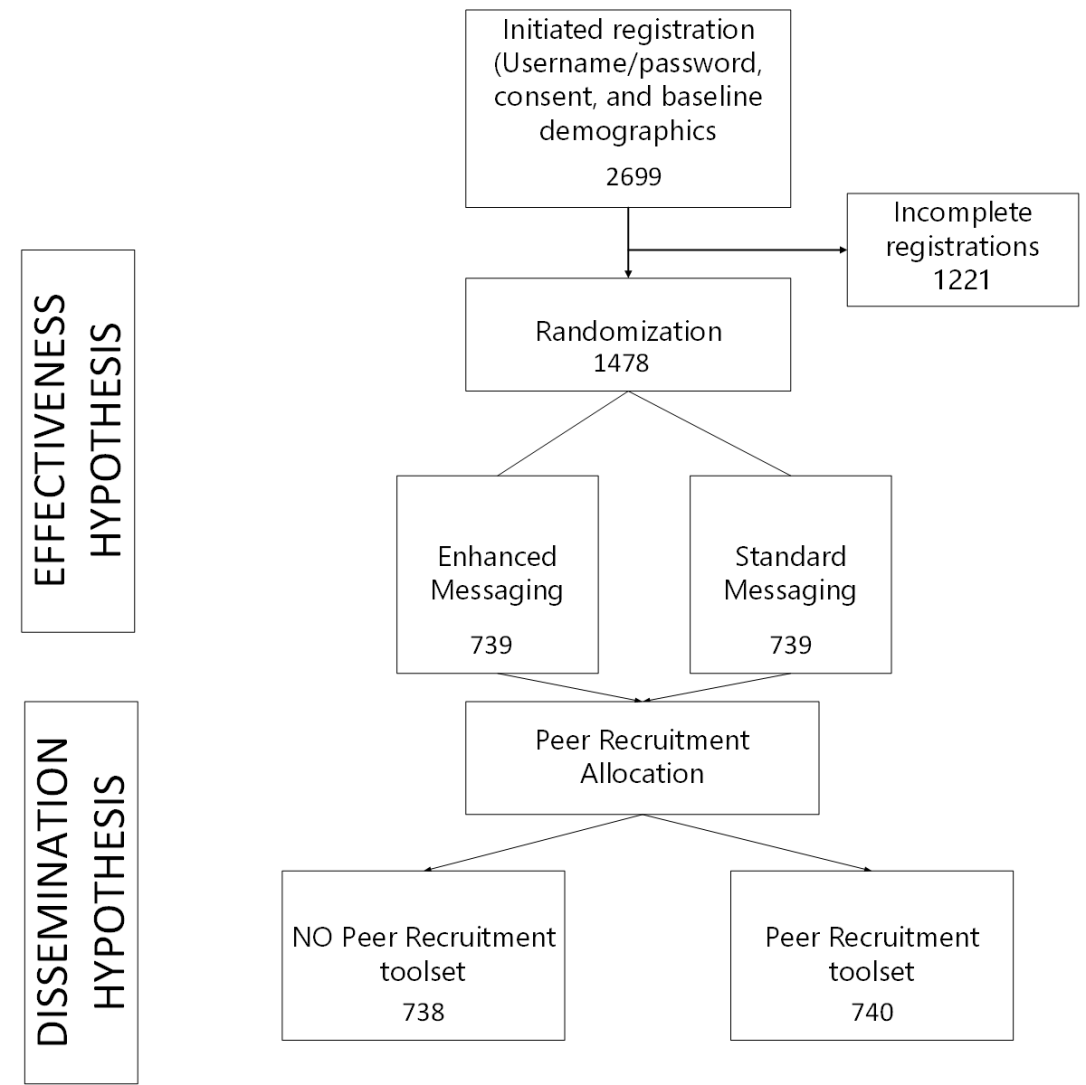
Flow of participant randomization. Allocation to receive the peer recruitment tools occurs in two phases or waves. Wave 1: Half of the enrolled smokers are randomized to receive peer recruitment tools, while the others do not receive the tools; and Wave 2: All subsequent smokers who are randomized to the effectiveness trial and also report they were peer-recruited are given access to the peer-recruitment tools.

The basic flow of a single hypothetical smoker through the peer-referral, registration, and subsequent initiation of new peer-recruitment is described in Figure 1. Peer recruitment will take place in waves. As is common in peer recruitment approaches, to initiate the waves, we will recruit the first wave (wave 0 or seeds) of smokers. Seeds will be recruited using online advertisements. Once a seed registers on Decide2Quit and receives the peer recruitment toolset, we expect the following to occur:

1. The seed consents to be in the study and recruit smokers from his/her network using the peer-recruitment tools (by sending a Facebook private message).
2. The successfully peer-recruited smoker (wave 1 recruit) registers on the system and consents to recruit other smokers in his/her social network.
3. The wave 1 recruit then continues the peer-recruitment chain, recruiting smokers his/her their social network.
4. The successfully peer-recruited smoker then registers (wave 2 recruit). The waves progress until the target sample size is reached.

### Study Design

As noted in Figure 1, all participants registering online on the Decide2Quit DISC will first undergo an effectiveness randomization and then a peer recruitment allocation. The effectiveness randomization will randomly assign participants to either receive access to the recommender system CTHC or the standard CTHC. The peer recruitment allocation will depend on the recruitment source. As noted in the section below, we will either directly recruit participants to the study (via search engine advertisements and research match) or they will be recruited by their peers (peer recruited). Directly recruited peers will be randomized to either receive the peer recruitment toolset or not receive the toolset. All peer recruited participants will receive access to the peer recruitment toolset. We are using this approach for the peer recruited smokers to enhance blinding. This decision was made because these recruited smokers may communicate with their peers about the intervention.

### Recruitment

#### Process

Our primary recruitment method will be to directly recruit smokers online using search engine advertisements or ResearchMatch. Participants having access to the peer recruitment toolset will be able to recruit their friends and family smokers to the website. To recruit via search engine advertisements, we will develop and post online advertisements customized to appear to smokers searching for quit smoking–related search terms online. When smokers click on these advertisements, they are redirected to Decide2Quit, where they are provided study information and registration instructions. The functions are provided in the ad managers of the search and social media website-targeted ads for smokers. For example, the Facebook ad manager allows advertisers to target users based on their interests derived from their profile’s keywords, pages they like, and groups they visit, which are then displayed on the Facebook page of the user. ResearchMatch is a free and secure online tool developed by Vanderbilt University and used by academic institutions across the country. To register, volunteers enroll on the ResearchMatch website, fill out demographic and optional health history questionnaires, and submit their profile. As noted, we will test peer recruitment for increasing access to the Decide2Quit DISC. Thus, we may also have participants who are peer recruited to the study as a consequence of our peer recruitment experiment.

#### Randomization

We will embed randomization within the technology. Our statistician will generate a randomization table; the randomization sequence will be conducted in random blocks of different sizes (n=8 and n=12) to ensure balance among the groups and reduce predictability of the allocation process. Thus, randomization will occur automatically at the time of initial registration. Following randomization, there will be an allocation to receive the peer recruitment toolset or not to receive the toolset. Study staff will be blinded to allocation during initial baseline assessment and follow-up.

### Study Participants

Participants will be included if they are current smokers over 18 years of age and can read or speak English. Prisoners will be excluded. Pregnant women may be incidentally enrolled. In such cases, this research poses no risks to the fetus.

### Data Collection and Outcomes

S2S will include multiple data collection stages (Table 1). At each time point (1 week, 1 month, and 6 months), we will send participants email reminders to complete the online follow-up surveys. We will send up to four email reminders over the course of 2 weeks from the targeted follow-up date (eg, if the participant is due for their 6-month follow-up on January 1, we will send them reminders for the 2 weeks following that date). If participants fail to respond to our email messages, we will call them to complete the survey over the phone. To calculate the success of our intervention, we will use the RE-AIM framework and evaluate results along the RE-AIM dimensions for success of health behavior interventions including reach, effectiveness, adoption, implementation, and maintenance [30-32]. Table 1 provides a list of study outcomes associated with RE-AIM dimensions. Our primary and secondary outcomes are listed below.

**Table 1:** Study measure by time points and associated RE-AIM dimensions.

Construct		Description	RE-AIM dimension
**Baseline measures**			
	Demographics	Age, gender, race, ethnicity, education level Contact Information	Reach
	Smoking habits	Current smoking habits	Reach
	Quit attempts	Readiness to quit Nicotine dependence Nicotine replacement therapy use E-cigarette use	Reach
	Family-based intervention and social network	Household status Smokers in social network Interest in family-based intervention	Reach
	Reach and registration	Number of users who saw and clicked on online advertisements Number of users who registered following online advertisements	Reach, Adoption
**1-month measures**			
	User feasibility and acceptability	User feedback on use of the system	
	Peer recruitment success	Number of peer recruiters Number of peers recruited	Reach, Adoption, Effectiveness
	Peer recruitment experience	Number of friend/family smokers a recruiter contacted for recruitment (network reached) Use of tools outside the Smoker-2-Smoker peer recruitment toolset Barriers/facilitators to peer recruitment Primary reasons a potential recruitee chose to be peer recruited or not	Reach, Implementation, Maintenance
	Perceived influence of peer recruitment on cessation	Beneficial to the participant’s quit smoking efforts Motivated to get support from those around the participant to quit smoking Increased the participant’s craving for cigarettes Made the participant feel like they were being helpful to their family and friends who are smokers	Effectiveness
**6-month measures**			
	7-day point prevalence smoking cessation	Do you currently smoke cigarettes (smoked even 1 puff in the last 7 days)? [33]	Effectiveness
	Biochemical verification of smoking cessation	NicAlert uses a dipstick to measure the level of cotinine in a sample of saliva. We will mail strips with instructions on how to take and return a picture of the results to us electronically.	Effectiveness
**Continuous measures**			
	Website engagement	Number of visits to decide2quit Number of pages used	Adoption
	Message feedback	Influence survey sent after each email (explicit)	Effectiveness

#### Effectiveness Outcomes

##### Smoking Cessation: 7-Day Point Prevalence and Verification by Saliva Test NicAlert

The primary outcome measure will be 7-day point prevalence at the 6-month follow-up. The 7-day point prevalence will be assessed by asking, “Do you currently smoke cigarettes (smoked even 1 puff in the last 7 days)?” [33] The 7-day window provides an appropriate stringent measure to account for a cross-sectional snapshot. In a cessation trial, biochemical verification is used to monitor for differential misclassification by the randomization group. The degree of misclassification is moderated by characteristics of the smoking cessation intervention [34]. Studies that are in-person and intense generally have more misclassification because of the personal connection between the smoker and the counselor and therefore require biochemical verification. Less misclassification occurs in low intensity, light-touch studies. Further, differential misclassification increases with intervention differences between two groups. Since our intervention and control are both texting interventions, the potential for differential misclassification is reduced. Further, requiring biochemical verification can, in fact, lead to additional issues, including refusal to participate, thus biasing the sample [34,35]. However, based on peer review, we are conducting biochemical verification. To reduce the potential for biasing the sample due to the need for biochemical verification, we are using an opt-in procedure to conduct biochemical verification among those who indicated they had quit smoking at 6 months. If a participant indicates they have quit smoking at 6 months, they are contacted by the research study staff to see if they would like to opt-in to the biochemical verification. If a participant opts in, they will be mailed the NicAlert test strips (Nymox Corporation, St Laurent, Quebec, Canada) within 24 hours with clear instructions on how to take a picture and return the picture of the results to us electronically. NicAlert is a semiquantitative method that uses a dipstick to measure the level of cotinine in a sample of saliva. The test strip displays the result in seven zones. Each zone represents a range of levels of cotinine/smoking (eg, zone 0: 0-10 ng/mL, a nonsmoker, zone 6: >1000 ng/mL, a heavy smoker). The results will be read as 0-6, and as recommended, any value ≥ 1 will be considered as tobacco use [36-38]. Our staff will also be available by phone to help the smokers complete testing. Participants sending back the sample will receive an additional US $50 incentive (as outlined in the consent form) for completing biochemical verification.

##### Risk Reduction (Reduction in the Number of Cigarettes Smoked)

We will calculate risk reduction by subtracting the number of cigarettes smoked at baseline from the number of cigarettes smoked at the 6-month follow-up.

#### Dissemination Outcomes

Secondary outcomes include recruitment rate, website engagement, and patient-reported outcomes collected via the 6-month follow-up questionnaire.

#### Recruitment Rate

When smokers register on Decide2Quit, they will be assigned a unique identifier and their registration date and time will be recorded. We will compute recruitment time from these data as the time taken to recruit each participant from the time that the first participant in the group was recruited.

#### Website Engagement and Feedback: Implicit and Explicit

We will use repeated use over other use measures (number of logins) to measure website engagement, as this has demonstrated an association with smoking cessation [39]. This is an ordinal scale of the number of Decide2Quit functions used after the first DISC visit (0: no functions used; 1: use of 1-2 functions, 2: >2 functions used). We will also continuously assess explicit (influence survey) and implicit (days to click on website) feedback after each email sent. When a smoker is sent an email, we will include a link to rate the message on the influence scale.

### Sample Size

#### Hypothesis 1: Dissemination

Our previous work [26,27] showed that having access to peer recruitment increased the proportion of African Americans to 23%, compared to 11% in the initial seeds (those recruited by advertisements). Using 10% as the base rate in the nonpeer recruitment group (no access to peer recruitment), we estimated sample size requirements by varying the proportion in the peer recruitment from 16% to 20%. With these assumptions, for our primary aim, we will need 219 smokers in each group to detect a difference of 10% (power=80%, α=.05). If we reduce the difference to 8% and 6%, we will need 319 and 525 participants in each group, respectively. Given that we will work with our panel to encourage recruitment of African American smokers in the peer recruitment group and may see bigger differences than those in our pilot study, we will have adequate power, particularly with the proposed sample size of 600 in each recruitment method. For recruitment time, previous trials estimated that the mean number of days to recruit a sample of 700 smokers was 244 (SD 81) days [27,40]. Assuming that peer recruitment proceeds with the same rate and SD, we can detect a difference in recruitment time as low as 14 days. Since we expect the comparison rate to be much slower, we are adequately powered to detect differences with a sample of 600 (power=80%).

#### Hypothesis 2: Repeated Use of Website

We used the method published by Whitehead to calculate power for this hypothesis [41]. In our previous work, we found a linear association between the 6-month cessation and repeated use by using the repeated use scale. For every increase by one in this scale, odds of smoking cessation increased (OR 2.10, 95% CI 1.03-4.30) [39]. With the current sample size of 300 per group, we can detect a difference a cumulative odds ratio of 1.7. Thus, our study is adequately powered to measure a reasonable difference in the repeated use measure.

#### Hypothesis 3: Effectiveness

We assumed a control cessation rate of 15% [42], and a two-sided significance level of .05. A sample size of 300 in each group will achieve 80% power to detect a difference of 9% (quit rate in intervention=24%) in quit rates between the two groups, based on a Z-test with pooled variance. We will categorize the NicAlert test results into smokers and nonsmokers and use the chi-square statistic to test for differences. We calculated the detectable difference in risk reduction with 300 smokers in each group and a mean of 3.3 cigarettes in the comparison group, using SDs of 2 and 3 with 80% and 90% power, respectively. We will have 90% power to detect a difference of 0.80 (or smaller) in the number of cigarettes smoked between the two groups. This difference is likely to be achieved based on the results of our PCORI pilot, in which we achieved a reduction of 0.85 (4.15-3.3) in 30 days compared to smokers receiving the standard CTHC messages; smokers receiving the recommender CTHC had a higher reduction in the number of cigarettes at 30 days (Standard CTHC: mean 3.3; S2S adaptive CTHC: 4.15).

### Statistical Analyses

All primary analyses will be conducted on an intent-to-treat basis. Secondary analyses will explore dose-response effects among those with variable levels of adherence to the intervention. All analyses will be two-sided, and the alpha error will be set at .05. We will begin our analysis by examining univariate statistics (means, medians, SDs, and 95% CIs) and distributions. We will examine the balance of participant characteristics by study groups and account for any imbalances in our multivariable analysis. As appropriate, differences in measured characteristics (ie, demographics and prebaseline smoking behaviors) by group will be tested using Chi-square tests of independence (categorical variables), analysis of variance (continuous variables), or the equivalent nonparametric tests, depending on the distribution of the variables. Differences in baseline characteristics of the intervention and comparison groups will be assessed.

To test Hypothesis 1, we will categorize the smokers as either African Americans or not, and then use the chi-square statistic to test for differences between the peer recruitment and standard groups. We will also compare mean recruitment time between the two types of recruitment method using a *t* test. We will explore possible factors that may not be balanced between the smokers recruited from the two methods. If we find any significant differences, we will develop a linear regression model to further adjust for the influence of the confounders on the time to recruitment outcome. Within the peer recruitment groups, we will conduct a secondary analysis examining differences in demographic characteristic between peer recruited and directly recruited smokers. Using data provided by search engine advertisement managers, we will evaluate the performance of our online advertisements (number of users registered on Decide2Quit following an advertisement on the search engine).

To test Hypothesis 2, we will use a generalized linear model, which includes indicators of peer recruitment and recommender CTHC and the interaction between the two indicators as independent variables.

To test Hypothesis 3 (effectiveness), we will compare participants randomized to enhanced CTHC and those randomized to standard CTHC. We will use the 7-day point prevalence cessation of the 6-month follow-up as the dependent variable in generalized linear models. Using mediation analysis, we will examine the potential mechanisms through which we anticipate the intervention to produce a beneficial effect. We will categorize the NicAlert test into smokers and nonsmokers’ categories and use the Chi-square statistic to test for differences. If risk reduction (decreased number of cigarettes smoked) is normally distributed, we will use the identity link function in the generalized linear model. We will also model risk reduction using count regression with a Poisson or negative binomial regression modeling if the variance of the distribution of risk reduction is over dispersed. For Hypotheses 2 and 3, we will compare African American smokers across the groups, and African American and white smokers for heterogeneity.

To assess the difference in smokers comparing the peer recruitment and standard recruitment, we will use a three-step strategy. First, we will collect data on covariates (minority status, education, readiness to quit, and income) that have been shown to differ between the peer recruitment and standard recruitment [26]. We will use these variables to adjust our overall models with the outcome of smoking cessation, comparing participants from peer recruitment and those from standard recruitment. Second, peer recruitment is, in many ways, analogous to clustering. Each person who is recruited by another individual is clustered in the group of the initial peer recruiter. Thus, some component of the difference is within the relationship between recruiter and recruitee. To address this issue, we included a marker for each “recruiter” as a fixed effect in the model. Third, we will use an advanced approach, termed complier-averaged causal effect analysis (CACE), to compare those who complied (peer recruited or not) in the intervention group with those who would have complied in the comparison group if they had been exposed to the intervention (peer recruitment). Thus, after adjustment using the first and second approach, we will conduct additional models using CACE.

For the budget impact analysis, we will compare the costs of the four intervention arms from the perspective of an implementing organization. The research team will track staff time associated with each intervention arm, including time for training, recruiting, and administering the different aspects of the intervention such as incentives for recruitment. In addition, we will work with the research team to estimate development costs of each intervention component (eg, the adaptive component of the CTHC) and any equipment or supply costs. We will compare the costs of the different intervention arms in multiple scenarios in which we examine how costs change based on changes in the components of the intervention and the types and amount of staffing provided for implementation. The economic analyses for will primarily consist of descriptive statistics. Using the estimates of costs of supplies, equipment, and staff time and the potential savings that result from decreased health care costs related to smoking cessation, we will calculate the budget impact of implementing a particular treatment strategy from the perspective of an implementing or disseminating organization. We will follow the guidelines outlined for best practices in budget impact analysis [43]. We will create tables to describe the assumptions of our inputs and outputs of our budget impact analysis and perform sensitivity analyses to examine how changing the assumptions of the model impact the potential costs for an organization implementing the intervention.

## Results

The project was funded in 2017, and enrollment will be completed in 2019. Preliminary data analysis is currently under way.

## Discussion

The S2S study addresses a key question raised in the State-of-the-Science Conference Statement on Tobacco Use: What are the effective strategies for increasing consumer demand for and use of proven, individually oriented cessation treatments, including among diverse populations? [5]. The primary goal of the intervention is to disseminate and increase the use of a tobacco cessation website using peer recruitment and enhanced CTHC. These methods will be compared to traditional online advertisements and standard CTHC. Although our pilot data are promising, we acknowledge that an effect size could have occurred due to chance and because the cessation results were limited to a short-term outcome (1 day). Thus, the present study is needed to detect if differences exist when fully powered while also examining a long-term (6-month) cessation outcome. We also identify the inherent challenge of measuring cessation outcomes by recruitment type for our dissemination hypothesis (testing the reach of standard versus peer recruitment). We have addressed this unavoidable challenge in our statistical analysis plan.

PCORI defines dissemination as the active and targeted approach of spreading evidence-based interventions to potential adopters and the target audience through determined channels using planned strategies, and its goals is to increase the reach of information, motivation, and patients’ ability to use and apply evidence [44-46]. Thus, both recruitment and use measures are needed to appropriately evaluate our DISC dissemination strategy. If recruitment is unsuccessful, the intervention’s reach is low. If recruitment is successful, but the intervention does not motivate repeated use, there is low intervention fidelity, which may reduce the patient’s motivation and ability to apply evidence. To ensure successful recruitment, we will continuously monitor these methods with our patient stakeholders to refine our advertisement strategies.

As access to the internet continues to grow, interventions like S2S will be increasingly accessible. It is important to continue to test methods to disseminate technology interventions to augment care for users. These technology interventions can serve as important augmentation for those receiving in-person and telephone counseling (to use between sessions and for longitudinal support). For those without access to other options, these technology interventions may serve as the only source of tobacco cessation support.

## Appendix

Multimedia Appendix 1 Patient Centered Outcomes Research Institute peer-review letter.

## Abbreviations

BPMF: Bayesian probabilistic matrix factorization
CACE: complier-averaged causal effect analysis
CTHC: computer-tailored health communication
DISC: digital intervention for smoking cessation
PCORI: Patient Centered Outcomes Research Institute
RCT: randomized controlled trial
S2S: Smoker-2-Smoker
