# Supplementary material for: Dissemination and Effectiveness of the Peer Marketing and Messaging of a Web-Assisted Tobacco Intervention: Protocol for a Hybrid Effectiveness Trial
Source: JMIR Res Protoc. 2019 Jul 23;8(7):e14814. doi: 10.2196/14814 (PMC6683651; doi:10.2196/14814)
Supplement: Multimedia Appendix 1 [file resprot_v8i7e14814_app1.pdf]

PATIENT-CENTERED OUTCOMES RESEARCH INSTITUTE
SUMMARY STATEMENT
(Privileged Communication)

**Principal Investigator:**	Rajani Sadasivam
**Organization:**	University of Massachusetts Medical School
**Project Title:**	Smoker-to-Smoker Peer Marketing and Messaging to Disseminate Tobacco Interventions
**PCORI Funding Announcement:**	Communication and Dissemination Research
**Review Cycle:**	2016: Cycle 1
**Request ID:**	R-1603-34645

AVERAGE OVERALL SCORE: 29

In-Person Review Discussion Notes:
Strengths:

- Reviewers expressed strong overall enthusiasm for the application and emphasized the strong potential for the proposed smoking cessation intervention to impact smoking, a highly significant public health problem and leading cause of death in the United States.
- Using peer marketing and machine learning techniques to disseminate an effective evidence-based digital intervention for smoking cessation (DISC) is highly innovative and a major application strength. Reviewers highlighted that, if successful, studying findings could have important applicability to other healthcare issues, such as weight loss and anxiety.
- Reviewers considered it is a strength that the intervention is free standing and does not depend on clinical practice or other healthcare system/infrastructure. Dissemination of the intervention is further supported by its minimal cost and self-supporting, self-propagating nature. The potential to link the intervention to the electronic health record environment further supports its dissemination capacity.
- Reviewers considered the application’s overall technical merit a major strength of the application. In addition to a strong study design and inclusion of rigorous methods, both the standard and enhanced Decide2Quit interventions are clearly described, appropriate to achieve the desired outcomes, and well validated.
- Finally, this resubmitted application reflects a number of important improvements, such as the inclusion of a budget impact analysis and the addition of two community-based stakeholders and a researcher with expertise in healthcare economics and outcomes. These were major strengths of the application.

Weaknesses:

- The inclusion of financial incentives to spur recruitment in the peer-to-peer intervention arm was considered by reviewers not only unrealistic for less-resourced organizations, but also inappropriate given the potential for participants to manipulate the recruitment process for financial gain. While a major concern, reviewers noted this weakness could be mitigated by removing the financial incentive or, potentially, by providing an evidence-based rationale for including the financial incentive. Rather than incentivize recruitment, reviewers suggested that study resources would be better allocated to enhance follow-up and minimize attrition, especially given the substantial risk of loss to follow-up.
- While the University of Massachusetts is currently supporting the intervention, the longer-term

durability of the resource is unclear; this minor weakness is fixable with further clarification in the application.
- Finally, some reviewers noted as a minor weakness that certain populations of interest and in need of smoking cessation support, in particular elderly adults who are potentially less digitally engaged, might not be reached by the online social media-mediated intervention.

Additional Comments:
Reviewers strongly commended the application's patient-centeredness, in particular the direct inclusion of smokers in the development of the intervention and the stakeholder-driven selection of patient-centered primary and secondary outcomes. Reviewers also commented that the application's framing of patient partners as thought leaders, as opposed to consultants, reflects a deep respect for patient partners. Despite these strengths, reviewers noted that the proposed engagement plan does not sufficiently describe the specific roles stakeholder will have throughout the study or explain why some stakeholders are compensated at lower rates than others. Further clarification and reconciliation of these minor concerns would strengthen the application.

Criterion 1: Potential for the study to fill critical gaps in evidence

Reviewer 1:

Strengths:

- The application addresses smoking cessation, with smoking constituting a leading cause of morbidity and mortality in the United States and worldwide.
- The application cites evidence from systematic reviews documenting uncertainty in the effectiveness of internet based interventions for smoking cessation as well as the impact of interactive features of such systems on such effectiveness. This is a major strength.
- The application cites evidence that suggests that effective web-based interventions in this area need to address both efficiency of recruitment and effectiveness of messaging to be maximally effective. This is a moderate strength.
- The proposed research will study the impact of both recruitment efficiency and tailored messaging in enhancing the likelihood of smoking cessation in users.

Weaknesses:

- None.

Reviewer 2:

NA

Reviewer 3:

NA

Reviewer 4:

Strengths:

- The applicants have demonstrated that they are targeting a public health concern, as both deaths caused by smoking and smoking-related diseases are leading contributors to mortality and morbidity. (Major)
- The applicants have found in prior studies that peer recruitment is especially effective over computer recruitment. Further, these techniques are even more effective within African American communities, who they demonstrate disproportionally higher morbidity and mortality as a result of smoking as

compared to other racial groups. This population also tends to seek cessation support less often than other racial groups, thus contributing to the significance of this proposed study. (Major)
- Previous research by the applicants has demonstrated the efficiency of S2S functions (peer recruitment and recommender computer tailored health communication) to disseminate Decide2Quit and improve its effectiveness. However, the applicants report that digital interventions for tobacco use have not been sufficiently tested. Given the findings of applicant's previous research in this realm, this provides justification for further investigation. (Major)

Weaknesses:

- The case for the study would be improved if the applicants provided background on strengths and weaknesses of standardized clinical treatments currently used for smoking cessation, and which are most commonly used by patients. This would further highlight the need for increased Digital Interventions, and could potentially speak to the acceptability and feasibility of these interventions. (Minor)

Criterion 2: Potential for the study findings to be adopted into clinical practice and improve delivery of care

<u>Reviewer 1</u>:

Strengths:

- The proposal is to validate the effectiveness of a web-based resource that combines user engagement in expanding recruitment of additional smokers with tailored messaging to the enrollees. This is a major strength.
- The dissemination plan provides for involving community-based organizations such as MOSAIC in promotional efforts and has engaged them in the development process. This is a moderate strength.
- The application provides for integration of referral options for the system into electronic health record (EHR) frameworks. This is a moderate strength.
- To the extent that the resource is both effective and independently supported, its use should be self-propagating, meaning that it will connect directly to patient users through enhanced recruitment. This is a major strength.
- The protocol will emphasize the potentially incremental effectiveness of the enhanced web-based approach in connection with African-American population, a particularly vulnerable one with respect to tobacco related disease. This subgroup is projected to constitute up to 20% of the enrolled subjects. The application furthermore provides evidence that this subgroup has a high level of access to and use of the internet. This is a major strength.

Weaknesses:

- The application includes a pledge of support for sustaining the resource on the part of the sponsoring institution, the Department of Quantitative Health Sciences of the University of Massachusetts. However, it is unclear how the long term support and financing is to be approached once the research agenda is complete. This was raised as a concern on the part of the earlier round of reviews and, despite bringing a health economist on board the research team, remains at least a minor weakness.
- The protocol provides for a financial incentive to users of $15 per additional recruit, with a maximum of seven reimbursable recruits. Since this incentive connects directly to a major outcome of the study and would not be expected to be provided to users outside of the research setting, its use potentially corrupts the validity of the recruitment results relative to a real world setting. This issue was raised in the first round of reviews but has not been sufficiently addressed in the revised application. This is a major, although easily remediable, weakness.
- Although the patient panel is cited as a vehicle for dissemination of findings of the study, it is not elaborated how they will go about this and what resources are available to them for this purpose. This is a minor weakness.
- The application appears to exaggerate the effectiveness of the enhanced CTHC component of the planned intervention as observed in the pilot studies by recurrently citing a 30% compared to a 20% rate

of reported cessation with no mention of statistical significance. In fact, the cited pilot study (citation #10) seems to report a maximum cessation rate of 26% compared to a control rate of 17% , a result that fell short of statistical significance. Marginal effectiveness of the enhanced intervention package could limit dissemination and adoption of the findings. This is a moderate weakness.

Reviewer 2:

Strengths:

- Smoking is the leading cause of preventable death in the United States. The effects of smoking are proven to be connected to chronic diseases of the lungs and heart. Yet this public health issue can only be tackled by the smoker and that knowledge is in part the core of Smoker-to-Smoker (S2S) Peer Marketing and Messaging to Disseminate Tobacco Interventions. If the study is successful, it can help the public, with an emphasis on an African- American demographic, to avoid health issues by choosing to stop smoking. It will decrease a public health concern. This is a major strength
- DISCs are health communications and accessible by computers and smart phones. The finding from this study could be reproduced by other dealing with other health issues (i.e., weight loss, anxiety) and could result in improvement in practice and patient outcomes. The only barriers related to the reproduction of using this study, since the research is producing a manual and is more than willing to share its results, is the inability to pull in recruits to the study. Otherwise, the study could be adopted for other health issues and this is a major strength.
- The study identifies appropriate end-users of this study as patients who are smokers who want to quit and have tried various times. Having digital access to information and support allows smokers to quit on their time. It offers motivation anytime day or night and addresses help with other concerns that arise from quitting (i.e., weight gain, nervousness). Since the messages are tailored to that person using data they have given, the messages are more personal and specific to his or her needs. That is a major strength.
- A research health scientist with experience in the area of heath economics and outcomes, has been added to the research team to replicate the intervention. This addition to the team comes in response to feedback received from PCORI reviewers during a previous submission of the study. This is a major strength.

Weakness:

- Letters of support from the National Cancer Society or American Lung Association would lend support to the importance of this study for public health. This is a minor weakness.

Reviewer 3:

Strengths:

- In addition to assessing patient outcomes, the study also includes measures of patient recruitment and patient use (i.e., long-term engagement). Both are important measures for future implementation efforts. This is a major strength of the study
- The study includes plans for a budget impact analysis, which will help the investigators to quantify the resources needed to successfully replicate the intervention. The analysis will include such factors as staff time associated with each intervention arm, administering different aspects of the intervention, and use of incentives for recruitment. The application plans to compare the costs of the different intervention arms in multiple scenarios and to include potential cost savings related to decreased healthcare costs association with cessation. This is a major strength of the study that will make it more likely that others will be able to adopt the intervention.
- The proposal contains a broad dissemination plan that includes multiple target audiences, venues, and types of dissemination communication strategies. The team has identified multiple methods for disseminating to all key stakeholder groups – patients, clinicians, advocacy groups, health system leadership, and local and national community organizations. This is a major strength of the study and makes large scale adoption of the findings more likely.
- The dissemination plan identifies a number of possible end-users of the study findings. These include

patients, primary care providers, healthcare systems, and community-based organizations. The dissemination plan also includes sample strategies for engaging these end users after completion of the study in order to impact replication. This is a major strength of the study.

Weaknesses:

- While the proposal includes the cost of recruitment incentives in the planned budget impact analysis, the inability of an organization to provide recruitment incentives is not discussed as a potential barrier to implementation, particularly for publicly funded community-based healthcare organizations that will be less likely to provide incentives. While the intervention may be effective for those it reaches, the inability to incentivize patients to recruit others into the intervention will likely limit the reach of the intervention under these conditions. This could mean that the intervention will be less impactful for publicly funded agencies, many of which serve a high proportion of smokers. The empirical support for the remaining components of the intervention to be tested suggests that this is a minor weakness, however.

Reviewer 4:

Strengths:

- The proposed research is an innovative project in applying bottom-up, "user-driven" techniques used by companies such as Amazon to increase websites' access and engagement in a healthcare setting, and as such, presents significant potential to improve accessibility and uptake of tobacco cessation tools in target populations, such as the African American community. (Major)
- Preliminary studies do indicate the possibility of sizeable uptake via processes of peer recruitment. The applicants report that in their previous NIH funded study, 75.4% of participants agreed that peer recruitment was beneficial to their efforts to quit smoking. In the past, researchers have experienced difficulty engaging African American patients, highlighting the importance of this proposed study. (Major)

Weakness:

- More could be done by the applicants to describe the intended post-study dissemination plan. What are the overarching dissemination goals of the project? Should digital interventions be combined with other treatments like CBT and smoking cessation medication (i.e. gum, the patch)? Are digital interventions intended to stand independently as their own treatments? (Minor)
- The application does not sufficiently cite patient, caregiver, or clinician demand for this treatment, which is a moderate weakness of this application. More information of previous studies of this intervention and patient demand for peer-referred digital interventions would significantly strengthen this application. (Moderate)
- The applicants do not adequately clarify who will use the study findings or the intervention method post-study. It is implied that the key investigator is developing relationships with several community organizations like MOSAIC, a "minority health focused grassroots organization," which will be collaborating in the process of disseminating any results from this study. However, this potential collaboration seems to be in a fledgling state, as no methods, timeline, or target groups are mentioned for dissemination of results after the study is completed. (Moderate)

Criterion 3: Scientific merit (research design, analysis, and outcomes)

Reviewer 1:

Strengths:

- The application cites self-determination theory as a construct of relatedness in support of the self propagating recruitment design but does not elaborate its role or relevance in any detail. This is a minor

strength.

- The application addresses a concern raised in the earlier review cycle regarding appropriateness of the 7-day point prevalence outcome measure and cites literature in support of the relevance of this outcome. This is a moderate strength.
- The interventions and comparators to be employed pertaining to both recruitment and effectiveness of messaging are described in detail and are well defined. The application addresses comments arising from the earlier cycle of review in this respect. This is a major strength.
- The protocol involves a 2X2 factorial design, which will enable independent analyses of the impact of the two components of the active S2S intervention, peer recruitment and the enhanced CTHC approach. This is an effective response to the previous round of reviews and is a major strength.
- The protocol describes a rigorous approach to power analysis, drawing on the results of pilot research, and loss of data. This is a major strength.
- The research team is headed up by individuals with extensive backgrounds of research in the target area who have worked together on many of the pilot studies. The team as a whole is multidisciplinary and multi-departmental within the University of Massachusetts family. This is a major strength.

Weaknesses:

- The investigation of heterogeneity of effect is largely limited to analysis of Caucasian compared to African American subgroups. This is a minor weakness.

Reviewer 2:

NA

Reviewer 3:

NA

Reviewer 4:

Strengths:

- The S2S condition is comprehensive and described in a thorough manner. The tools are varied and include a Facebook app, an online training video, and a recruitment tracker. (Major)
- Study outcomes address both dissemination and effectiveness measures. The 7 day point prevalence cessation measure, NicAlert, is biochemically verified and arguably more reliable than self-report measures of smoking cessation. (Major)
- The reduction in the number of cigarettes smoked is appropriate for the targeted population. (Major)
- The online format may come as the only source of tobacco cessation support for patients who find in-person counseling unavailable. This aspect of the study has the potential to significantly increase dissemination in target and at-risk populations. (Major)
- The applicants fully define the characteristics of study participants, especially as related to race, a central point of the study, as well as comparators and outcomes. (Moderate)
- The study plan seems highly feasible in terms of recruitment, as the applicants cite previous studies demonstrating the efficacy of "state-of-the-art" online recruitment strategies. Recruitment consists of a Facebook app, an online training video, and a recruitment tracker. Recruitment is set to occur in waves in order to benefit from the influence of peer recruitment, which has proven particularly effective with the African-American community, the target population of this study. In the applicants' prior NIH-funded study, the sample of 190 smokers was quadrupled within one year due to peer recruitment, thus empirically demonstrating the effectiveness of this protocol. (Moderate)
- The sample size projects 319 and 525 participants in each group respectively in order to detect difference of under 10% (power = 80%, alpha=.05) and as the applicants propose to recruit 600 participants within each group, the power estimates are appropriate. (Moderate)

Weaknesses:

- The applicants report that using online advertisements is the demonstrated "state-of-the-art" approach

to recruit smokers. However, this does not take into account smokers who are illiterate or who are not fluent English speakers. (Minor)
- The specific procedure for measurements of risk reduction was unclear. How will the repeated use ordinal scale, assessing the number of functions after participants' first visit to Decide2Quit, be recorded? Will it be function of the website, or will participants fill it out in person/in the presence of the researcher? These are important considerations regarding the validity of this outcome measures. (Minor)
- The challenge of attrition rates within the dissemination of DISCs is not thoroughly described. Applicants cite a generally high attrition rate, but do not note the specific figure, the causal factors behind attrition in DISC studies, and applicants provide an insufficient explanation of the ways in which they seek to approach this challenge to reaching an appropriate sample size. (Major)

Criterion 4: Patient-centeredness

Reviewer 1:

Strengths:

- The application reflects an extensive background of development, including multiple pilot studies, that forms the basis of the proposed trial. Patient and consumer inputs into all aspects of the background work have been extensive and compelling. This is a major strength.
- The application attests that outcomes such as "number of cigarettes" smoked have been found to be very important to patients in the course of the pilot work. This is a major strength.
- The protocol provides for patient-reported outcomes in a fashion appropriate to the design. This also is a major strength.
- The nature of the recruitment and recommender programs to be tested ensures substantial input from both the users and others enrolled in the program in shaping the content of the interventions. This is a major strength.
- Decide2Quit is an established smoking cessation website and is currently available to consumers as an educational site with interactive features. This is a moderate strength.

Weaknesses:

- None.

Reviewer 2:

Strengths:

- The project's 2S2 recruitment process has five patients on their panel, including one African-American and is working to recruit another in response to previous feedback from PCORI reviewers. The study team will continue to look for more recruits to participate in the study; the changes in recruitment strategy are positive and a major strength.
- In a previous NIH-funded study, the Facebook app, online training video and a recruitment tracker (toolkit) were given to recruits, and most participants strongly agreed that the peer recruitment, which included that toolkit, assisted them in giving up smoking. That speaks highly of previous successes and is patient-centered. This is a moderate strength.
- Baed on previous feedback, the applicants have added an African-American smoker and two members from MOSAIC Cultural Complex, a minority-health-focused grassroots organization as stakeholders. This is a major strength.
- Since smoking is the number one preventable cause of death in the United States, helping patients who want to quit smoking is of utmost importance. Dissemination of information, motivational messages, an online community and peer support through computers and smart phones give patients a voice and a choice in their healthcare outcomes. This is a major strength.

Weaknesses:

- While the change in recruitment makes room for additional patient participants of color, the statement of looking for "star" recruits is a bit unclear and limiting. This is a minor weakness.

- Using digital and online advertisements for recruitment comparison may be the state-of-art approach to recruit some smokers; however, since the target age is older than nineteen, there are many older and minority smokers who have smoked for decades but will not see the advertisements. The choice to not advertise in weekly free newspapers and senior or VA newsletters could mean a loss of many smokers who want to quit smoking but have no support. While this group of smokers often use cell phones and computers, and could participate in the study, they may not pay attention to advertisements. This is a minor weakness.

<u>Reviewer 3</u>:

Strengths:

- The two patient outcomes chosen for this study are smoking cessation and smoking reduction. The research team cited three sources of evidence demonstrating the importance of these two measures to patients who smoke. These were: (1) patient input from their previous studies, (2) confirmation from the current project's Patient Panel, and (3) focus group results from a recent study demonstrating that the outcome of most importance to smokers was risk reduction (rather than cessation). This is a major strength of the proposal.
- The study seeks to close the evidence gap on which strategies are effective for increasing consumer demand for and use of proven, individually oriented cessation treatments, including among diverse populations. This question was raised in the NIH State-of-the-Science Conference Statement on Tobacco Use, demonstrating support from a wide range of clinical stakeholders for studies that seek to answer this question. In addition, the investigators have benefited from extensive assistance from patients in the development of the specific intervention to be tested and its messaging system, suggesting that patient stakeholders also deem this type of study to be of value. Finally, the project has the support of local community minority health educators, also showing that answering research questions about how to best disseminate evidence-based cessation treatment strategies to diverse populations is an important endeavor. This is a major strength of the proposal.
- The Decide2Quit intervention is publicly available online at no charge. The intervention is widely available through a computer or smart phone. The investigators provide evidence of widespread access to internet and smart phones within the general population and among vulnerable populations, demonstrating the accessibility of the intervention and the appropriateness for inclusion in the study. Prior studies have demonstrated that repeated use of its functions is significantly associated with smoking cessation at 6 months. Research has also provided empirical support for the other intervention components to be tested – peer recruitment and computer tailored health communication. This is a major strength.

Weaknesses:

- None.

<u>Reviewer 4</u>:

Strengths:

- The applicants cited the fact that peer recruitment is an effective incentive for engagement, and as a previous study successfully engaged 759 participants, quadrupling the number of participants originally enrolled via peer recruitment strategies. In a smoking cessation program, such significant engagement implies substantial interest from the target population, although there is no direct evidence due to the fact that the majority of smokers generally do not express their wish to quit to health care providers. (Major)
- The Recommender CTHC personalizes messages based on smoker's feedback, creating tailored and

persuasive messages in response to individuals' beliefs, thus centering care on patients within treatment. Researchers cite the effect of the CTHC messages as more pronounced within African American participants and increasing DISC use. (Major)

- The proposal addresses one of the key questions mentioned in PCORI's definition of patient-centered outcomes research. Namely, "What can I do to improve the outcomes that are most important to me?". Specifically, the proposal aims to incorporate a wide variety of settings and diversity of participants to address individual differences and barriers to implementation and dissemination, namely access to intervention. (Major)
- A patient panel noted reduction in the number of cigarettes smoked as the most meaningful outcome, highlighting the patient-centeredness of this application. (Moderate)
- In addition to employing concepts and theories from several fields of research, applicants involved patients as content experts in the development of several of the S2S tools. (Moderate)

Weaknesses:

- There lacks a specific indication of how a Digital Intervention would be used in a clinical capacity in regards to supplementary cessation tools patients may be using simultaneously. For example: would Digital Interventions be intended for use alongside traditional smoking treatments like the patch or nicotine gum? Or is it intended to be the sole treatment? (Moderate)
- The proposal does not indicate evidence of an explicit patient desire for peer-referral Digital Interventions, with the exception of citing past success with peer referral programs. (Moderate)
- Applicants should further describe how they intend to target the majority of smokers who often lack an expressed motivation to quit. Even smokers who are highly motivated to quit often fail in these attempts, so motivation seems to present a tall barrier to success. The application should more thoroughly explain how peer recruitment successfully targets smokers expressly uninterested in quitting. (Minor)

Criterion 5: Patient and stakeholder engagement

Reviewer 1:

Strengths:

- The project will make use of a patient advisory committee (PAC) made up of several current smokers, including some African Americans. This is a moderate strength.

- Members of the PAC will be reimbursed for their time at a reasonable hourly rate. This is a moderate strength.

- Members of the PAC and of collaborating community organizations will be engaged to disseminate awareness of the Decide2Quit resource and the findings of the trial. This is a moderate strength.

Weaknesses:

- The names, backgrounds, and roles of the PAC members are not directly listed in the research plan, but are only mentioned in the abstract and, indirectly, via letters of support. This is a minor weakness.
- The research team does not include a consumer or patient as a co-investigator. This is a moderate weakness.
- The application does not make clear the extent that the patient collaborators or prospective PAC members were specifically involved in the design of the proposed trial, including choice of outcomes recruitment messaging etc. This is a moderate weakness.

Reviewer 2:

Strengths:

- The proposal includes letters of support from a grassroots organization whose mission is in part to improve quality of life for vulnerable populations. One of the co-founders of MOSAIC will participate throughout the study and is especially excited about the communication and dissemination of the tools developed. Their community engagement is an asset to the study and will help reach a vulnerable population that otherwise might not be reached. This is a major strength.
- Other stakeholders are patients who have either recently stopped smoking after decades or are still hoping to stop. They are enthusiastic participants who will help educate the researchers about the tools needed, the support needed and the emotional support necessary to help in smoking cessation. Another stakeholder who smoked cigarettes for more than 40 years is enthusiastic about taking part in the study and to help other through the dissemination process. In this resubmission, patients have been added to the dissemination process, which was part of the feedback suggested by previous reviewers. The addition of personal experience to this proposal is also an addition suggested by previous PCORI reviewers and shows the level of commitment these stakeholders have to participate. This is a major strength.
- The study compensates patient partners $50 per hour for their time and panel participation, which exhibits a respect for their participation and feedback. This is a major strength.
- The Advisory Board has the ability to make decisions about the study in all phases from recruitment to dissemination and includes patients working with community leaders, counselors and researchers. This is a major strength.

Weaknesses:

- Feedback and input from the two members of the grassroots organization will likely help reach African-American smokers and aid in the success of the smoking cessation program on all levels. While this is most likely a satisfactory agreement made between the members and the researchers, it is unclear why the study would compensate them less than a patient stakeholder. This is a moderate weakness.

Reviewer 3:

Strengths:

- Expertise of the research team is wide and varied and includes health disparities research, budget impact analysis, tobacco treatment research, tobacco treatment provision, computer engineering, and behavior change specialists. The team will also include five patients, two community leaders from a grassroots organization focused on minority health, and one tobacco cessation clinician. Collectively, these individuals fulfill the roles of researchers, patients, clinicians, and advocates. This is a major strength of the proposal.
- Scientists appear to have been engaged with their patient and clinical stakeholders throughout the process. Stakeholder partners specifically impacted outcome selection, creation of study tools, study design and refinement, and identification of dissemination venues. Examples include: (1) the identification of both the primary and secondary outcomes of the study by the patient partners, to include the recommendation to measure reductions in smoking in addition to smoking cessation, (2) development of motivational message language for messaging system, and (3) modification of recruitment strategies. This is a major strength of the study.
- The roles of the study partners are clearly specified. The Patient Panel (which also includes a clinician and representatives from a local minority health community-based organization) will assist with refining the online recruitment strategy (particularly for recruiting African American smokers), monitoring study progress, solving problems related to slow recruitment should they arise, evaluating data, helping to present results to smokers and community-based organizations, and assisting with additional dissemination efforts beyond the scope of the study period. This is a major strength of the study.
- The Patient Panel is empowered to serve as decision leaders rather than as mere participants or consultants. The proposal outlines a specific methodology – structured nominal group technique (NGT) process – that will be used to prioritize and establish decisions. This is a major strength of the study.
- The research setting is well suited and resourced for the proposed project. The university has extensive experience in conducting NIH-funded studies and houses a Center for Clinical and Translational Science and a Special Resource Population Center. Faculty and staff also have access to resources in health informatics, biomedical informatics, a clinical data warehouse, and a technology usability lab.

This is a major strength of the study.

Weaknesses:

- None.

Reviewer 4:

Strengths:

- The study team is multidisciplinary and has extensive prior experience collaborating. The team consists of computer engineers and clinicians, a health economist, as well as experts in behavior change, tobacco cessation, and health disparities, in addition to patient partners. (Major)
- Patients will be formally engaged as research consultants. The proposed patient panel will include five patients (including African American smokers), two leaders of a community-based organization focused on minority health, and a practicing tobacco treatment specialist. Patients will participate as decision leaders and content experts. They will be involved in the refinement of the recruitment strategy, monitoring the implementation progress, evaluating the data, etc. (Major)
- Patients developed several of the S2S tools as decision leaders and content experts. (Major)
- The applicants extensively described the selection process for patients chosen as research consultants. (Moderate)

Weaknesses:

- Only two of the five patient consultants are planned to be picked from the African American community, which may not provide a sufficient diversity of opinion within smoking cessation among African Americans, a central focus of this study. (Minor/Moderate)
- The decision making authority is unclear among the collaborators in this study (roles and duties are set to be decided in the first meeting, but a description should be provided before the initiation of the study). (Minor)

Overall Comments

Reviewer 1:

The application describes rigorous testing of effectiveness of a web-based resource that carries the compelling potential of facilitating distribution and use of a low-cost preventive healthcare intervention to vulnerable populations and to help mitigate the disease burden caused by smoking for such populations. The application also reflects extensive development and piloting efforts of the investigator team over a ten year period and enjoys the support of the sponsoring academic institution that has served as the home for these research and development efforts as well as sponsored the development and maintenance of the online resource itself for this period of time.

The application incorporates substantial responses to the prior round of reviews which include substantive changes in the research approach, such as the 2X2 factorial trial design, addition of co-investigators, clarification of the high level of access to and use of the internet by the vulnerable African American population that has been targeted for special attention in the course of the analysis. Strengths of the revised application include a very strong research design and a high degree of patient-centeredness. In the latter regard, both the nature of the interventions and of the approach taken to their development and piloting by the investigator team contribute to a uniquely patient-driven product. The application has adequately addressed prior reviewer concerns in this area.

The greatest weakness of the proposal, however, is in the area of patient and consumer engagement. Although the application identifies several individuals, including African Americans, who will serve as part of a patient advisory group in the course of the trial, it does so only marginally through inclusion of their letters of support

and stops short of incorporating them into the research team proper. It is therefore unclear what if any role if any they have played in shaping the specifics of the current proposal and is also unclear exactly what role they will be playing as the project proceeds. Hence the application can benefit from additional improvement in this respect. Another important, if remediable, weakness is the provision of a financial incentive to enrollees for recruitment of others is problematical. Such an incentive might be justified to the extent that their participation in compliance with the trial requirements for outcome assessment would be understandable. However, as an incentive to directly enhance a key outcome, i.e., peer recruitment, such an incentive opens the trial up to opportunism and poses a threat to the integrity of the results in this area. The limitation of reimbursable recruitment to seven additional enrollees does not adequately mitigate this threat, which was also noted in the course of the previous review cycle.

Other potential weaknesses include an apparent overstating the observed effectiveness of the intervention in pilot studies. If the current trial does not show a dramatic effect, particularly in the African American sub-group of enrollees, this will limit dissemination and uptake. The assessment of heterogeneity of effect is limited to discrimination between racial and ethnic subgroups and will apparently not address the importance of other subject characteristics in determining the outcomes. Finally, one issue raised by prior reviewers has only been partially addressed. Namely, some thought has to go into what will be required to maintain the availability of the online resource and the durability of the approach should the sponsoring institution be unable or unwilling to do so at some point in the future.

<u>Reviewer 2</u>:

The plan to disseminate an evidence-based Digital Intervention for Smoking Cessation (DISC)is being undertaken by a dedicated and knowledgeable team comprised of researchers, mental health counselors, patients, physicians and community leaders of vulnerable populations which would most benefit from the tools available, is in the interest of public health. It is patient-centered and shows enthusiasm and dedication from patient stakeholders. Reaching out to the African-American community, which has been documented to develop health issues from smoking at an increased rate after smoking for fewer years compared to other groups, is extremely important to the individuals, to health providers, and for public health. The willingness of the study team to help others to replicate the study is admirable and could help individuals who struggle with other health issues, such as obesity and anxiety. With past research results that show this toolkit helps people who wish to stop smoking, the study is patient-centered and important.

<u>Reviewer 3</u>:

Smoking has been cited as the number one preventable cause of death, yet the majority of smokers are not interested in quitting at any given time. Consequently, there is a need to improve methods of disseminating interventions that are easily accessible, have a wide reach, motivate long-term engagement of the end-user, and are culturally relevant for diverse populations. The proposed project provides an opportunity to test a combination of recruitment and engagement strategies for an evidence-based Digital Intervention for Smoking Cessation (DISC), with an emphasis on outcomes for African American patients who are at increased risk for adverse outcomes related to smoking. The proposal contains a number of major strengths in the areas of patient-centeredness, patient and stakeholder engagement, and potential for findings to be adopted into practice. These strengths include health-related outcomes of importance to patients, important implementation-related outcome measures, a budget impact analysis to inform future end-users about quantifiable costs related to implementation of various replication scenarios, strong patient and stakeholder engagement, and a broad and varied dissemination plan. The only weakness in these areas is that the proposal does not address the inability of an organization to provide recruitment incentives as a limitation to future replication efforts, particularly in publicly funded healthcare environments. The existing empirical support for the remaining intervention components to be tested, in combination with the numerous strengths of the proposal, renders this a minor weakness.

<u>Reviewer 4</u>:

Smoking is the number one preventable cause of death in the U.S., and estimates suggest that a significant proportion of USA health care expenditure is attributable to smoking. Additionally, African Americans are disproportionately affected by tobacco and their tobacco use directly leads to a variety of severe health issues. Thus there is clear need to address health disparities via improved dissemination methods for tobacco

interventions. The applicants provide a compelling intervention, as Digital Intervention for Smoking Cessation (DISCs) as an effective, but underused method. These health communication programs are highly accessible and have the potential to reach a larger and diverse group of smokers. Additionally, this project provides an opportunity to test the peer marketing model that has been successfully used by companies such as Amazon and Netflix, but has not been substantially researched in the realm of healthcare. This is a well-designed and methodologically rigorous study that is in line with PCORI's standards and goals. The applicants have sufficient background in the topics to be studied, as evidenced by a well-organized and meticulous procedure and dissemination plan. Additionally, applicants demonstrate the potential for patient engagement and patient-centeredness.

The applicants have addressed recommendations from previous reviewers, which have strengthened this resubmission overall. Recommended changes consisted of changing the design to a 2x2 factorial design. This change will allow the researchers to piece apart the effects of the two intervention components: recruitment strategy and recommended vs. standard intervention. Further, the applicants highlighted how they will increase dissemination to racial minorities, namely African Americans, by reviewing extant literature and including African American stakeholders in their patient panel. Third, the applicants highlight how the messaging tailoring strategies target patients and increase utilization. Lastly, the applicants present new data on ways to target minorities.

The most significant weakness of the application is insufficient evidence of information on patient demand. This weakness could potentially be mitigated by further discussion of how/if patients have asked for this type of intervention, or how patients have identified DISCs as a useful tool for smoking cessation. Further, there is need for a discussion of the ways in which study findings to be integrated into clinical practice.

On the whole, the applicants present a well-written proposal with a strong empirical support. Some areas of the application require additional clarification and elaborated discussion; however, the applicants present a meticulous, novel proposal with great potential for improving current smoking cessation methods and alleviating the substantial current health-costs burden of smoking-related disease and mortality.

Does the application have acceptable risks and/or adequate protections for human subjects?

Reviewer 1: Yes

Reviewer 2: Yes

Reviewer 3:

Reviewer 4: Yes

The application has acceptable risks to human subjects.

The applicants provide adequate remuneration for study participation, as will those smokers who sit on the patient panel ($50/hour for 10-15 a year). As mentioned above, the applicants do not describe how they will collect risk reduction data. However, all data collection methods are designed to protect confidentiality and patient privacy by utilizing the UMass Regulated Environment. The risks in this study are not high, and primarily include mild stress associated with completing the follow-up survey. Nevertheless, the applicants have taken adequate steps to protect against risk, including the use of random numbers to mask participant data and implementation of a protocol in the event that health risks arise. The applicants have also implemented a DSMB that will provide ongoing monitoring of the safety of subjects. In this case, the potential gains for this study outweigh the risks.
